# Increase in the Length of Lung Cancer Patient Pathway Before First-Line Therapy: A 6-Year Nationwide Analysis From Hungary

**DOI:** 10.3389/pore.2021.1610041

**Published:** 2021-12-23

**Authors:** Zoltan Kiss, Krisztina Bogos, Lilla Tamási, Gyula Ostoros, Veronika Müller, László Urbán, Nóra Bittner, Veronika Sárosi, Aladár Vastag, Zoltán Polányi, Zsófia Nagy-Erdei, Kata Knollmajer, Máté Várnai, Balázs Nagy, Krisztián Horváth, György Rokszin, Zsolt Abonyi-Tóth, Zsófia Barcza, Judit Moldvay, Gabriella Gálffy, Zoltán Vokó

**Affiliations:** ^1^ MSD Pharma Hungary Ltd., Budapest, Hungary; ^2^ National Korányi Institute of Pulmonology, Budapest, Hungary; ^3^ Department of Pulmonology, Semmelweis University, Budapest, Hungary; ^4^ Matrahaza Healthcare Center and University Teaching Hospital, Matrahaza, Hungary; ^5^ Department of Pulmonology, University of Debrecen, Debrecen, Hungary; ^6^ Faculty of Medicine, University of Pécs, Pécs, Hungary; ^7^ Center for Health Technology Assessment, Semmelweis University, Budapest, Hungary; ^8^ RxTarget Ltd., Szolnok, Hungary; ^9^ Department of Biomathematics and Computer Science, University of Veterinary Medicine Budapest, Budapest, Hungary; ^10^ Syntesia Medical Communications Ltd., Budapest, Hungary; ^11^ 1st Department of Pulmonology, National Korányi Institute of Pulmonology, Semmelweis University, Budapest, Hungary; ^12^ 2nd Department of Pathology, MTA-SE NAP, Brain Metastasis Research Group, Hungarian Academy of Sciences, Semmelweis University, Budapest, Hungary; ^13^ Pulmonology Hospital Törökbálint, Törökbálint, Hungary

**Keywords:** lung cancer, patient pathway, time to treatment, episode of care, system interval

## Abstract

**Objective:** This study aimed to examine the characteristics of the lung cancer (LC) patient pathway in Hungary during a 6-years period.

**Methods:** This nationwide, retrospective study included patients newly diagnosed with LC (ICD-10 C34) between January 1, 2011, and December 31, 2016, using data from the National Health Insurance Fund (NHIF) of Hungary. The following patient pathway intervals were examined: system, diagnostic and treatment interval by age, gender, tumor type, study year and first-line LC therapy.

**Results:** During the 6-years study period, 17,386 patients had at least one type of imaging (X-ray or CT/MRI) prior to diagnosis, and 12,063 had records of both X-ray and CT/MRI. The median system interval was 64.5 days, and it was 5 days longer among women, than in men (68.0 vs. 63.0 days). The median system interval was significantly longer in patients with adenocarcinoma compared to those with squamous cell carcinoma or small cell lung cancer (70.4 vs. 64.0 vs. 48.0 days, respectively). Patients who received surgery as first-line treatment had significantly longer median system intervals compared to those receiving chemotherapy (81.4 vs. 62.0 days). The median system interval significantly increased from 62.0 to 66.0 days during the 6-years study period.

**Conclusion:** The LC patient pathway significantly increased in Hungary over the 6-years study period. There were no significant differences in the length of the whole LC patient pathway according to age, however, female sex, surgery as first-line treatment, and adenocarcinoma were associated with longer system intervals.

## Introduction

Lung cancer (LC) is the most frequently diagnosed cancer and the leading cause of cancer-related mortality worldwide, with an estimated number of 2.1 million new cases and 1.8 million deaths annually in both sexes combined (1). Lung cancer affects both males and females, and it is especially prevalent in developing, middle-income countries (2). According to GLOBOCAN, age-standardized incidence rates in Central and Eastern Europe were 49.3 among males and 11.9 among females in 2018, which were the third highest rates globally (3). Hungary has traditionally been reported to have the highest lung cancer incidence and mortality in Europe, however, a recent study revised the available data and demonstrated that the country ranked third in terms of LC epidemiological rates (4,5,6).

Numerous publications have confirmed that lung cancer has one of the poorest 5-years survival rates of all cancers, ranging from 16 to 23% in all stages combined (7,8,9). One, if not the main, reason for poor lung cancer survival may be late diagnosis. Since the disease is often asymptomatic in early stages, lung cancer is diagnosed primarily in advanced stages. Therefore, patient access to first-line medical care is often delayed, and there may be long referral times from diagnosis to treatment within the patient pathway (10). The management of lung cancer has been affected by two parallel processes during the past few years. On one hand, the complexity and infrastructural requirements of lung cancer care have increased as more and more diagnostic and treatment options have become available (7). On the other hand, a slow but steady shift towards outpatient and community-based care has occurred in the developed world recently, with fewer services requiring lengthy inpatient hospital stays (7). Approximately since the turn of the millennium, the conventional approach of referring patients to multiple specialists in a sequential manner has become less acceptable in several countries, since this form of care is often slow, fragmented and poorly coordinated (11). Hence the in-depth analysis of the patient pathway of LC patients is of paramount importance in order to detect “bottlenecks,” and to serve as an evidence-base for healthcare policy.

Although Hungary is among the countries with the highest lung cancer incidence, the characteristics of the Hungarian lung cancer patient pathway have not yet been explored. Therefore, the aim of our study was to develop a method for measuring the phases of LC care within the patient pathway in Hungary, and to compare the results by age, sex and study years based on data from the National Health Insurance Fund (NHIF) database. Furthermore, we also aimed to identify any existing differences between the main Hungarian regions to provide a potential basis for more optimal healthcare resource allocation.

## Materials and Methods

The protocol of our study was approved by the National Ethical Board for Health Research (10338-5/2019/EKU).

We conducted a retrospective, longitudinal study using data from the National Health Insurance Fund (NHIF) of Hungary. The NHIF is a nationwide health insurance system which covers almost 100% of the Hungarian population and collects specific information regarding all in- and outpatient visits, including patients’ ID and ICD-10 codes, as well as prescriptions of reimbursed drugs. Furthermore, the NHIF database records the exact dates of all interventions as well as their recorded ICD-10 codes.

The target population of our investigation included patients newly diagnosed with lung cancer (ICD-10 diagnosis code: C34) between January 1, 2011, and December 31, 2016. Further inclusion criteria were: 1) age above 20 years at the time of diagnosis; 2) a minimum of two occurrences of the ICD-10 code C34 within more than 30 but less than 365 days, except if the patient had died within 60 days after the first C34 code, in which case only one occurrence of C34 was also accepted. Criterion 3) was set to avoid the potential miscoding of lung cancer and ensure that only patients truly involved in lung cancer care would be included. Patients with cancer-related ICD-10 codes other than C34 as well as those receiving anticancer therapy other than lung cancer treatment within 6 months before or 12 months after the first occurrence of C34 were excluded. The 3-years period between 2008 and 2010 was considered as reference period for the detection of newly diagnosed lung cancer patients after 2011. Patient population was stratified by age, sex, histology of lung cancer (where it was available), type of first-line treatment, study years and main Hungarian regions. Age cohorts were defined based on patient age at the beginning of study period, while mean age was calculated based on patient age at diagnosis.

Patient ID-based information was collected regarding diagnostic and therapeutic interventions, X-ray, CT or MRI, biopsy and first-line treatment within the investigation period of 120 days before and 90 days after the date of the first record of C34. Patients receiving first-line therapy for lung cancer were classified based on available data regarding diagnostic and treatment interventions. Several lung cancer related therapeutic options were recorded as first-line interventions (Supplementary Table S1). **Group A** comprised patients who had only one type of diagnostic imaging raising the suspicion of lung cancer (X-ray or CT/MRI), and first-line therapy codes. This group formed the basis for our further analyses. Patients with both types of diagnostic imaging (X-ray and CT/MRI separately) before the date of the first record of C34, and data on first-line therapy constituted **group B**. Patients with available X-ray, CT, and exact dates of biopsy, as well as first-line treatment codes were classified as **group C**. However, this population was significantly underrepresented in the original lung cancer population, therefore, we did not perform patient pathway analysis in this group. Nevertheless, we examined the distribution of time periods between the first C34 code and date of biopsy as shown in Figure 2.

**FIGURE 1:**
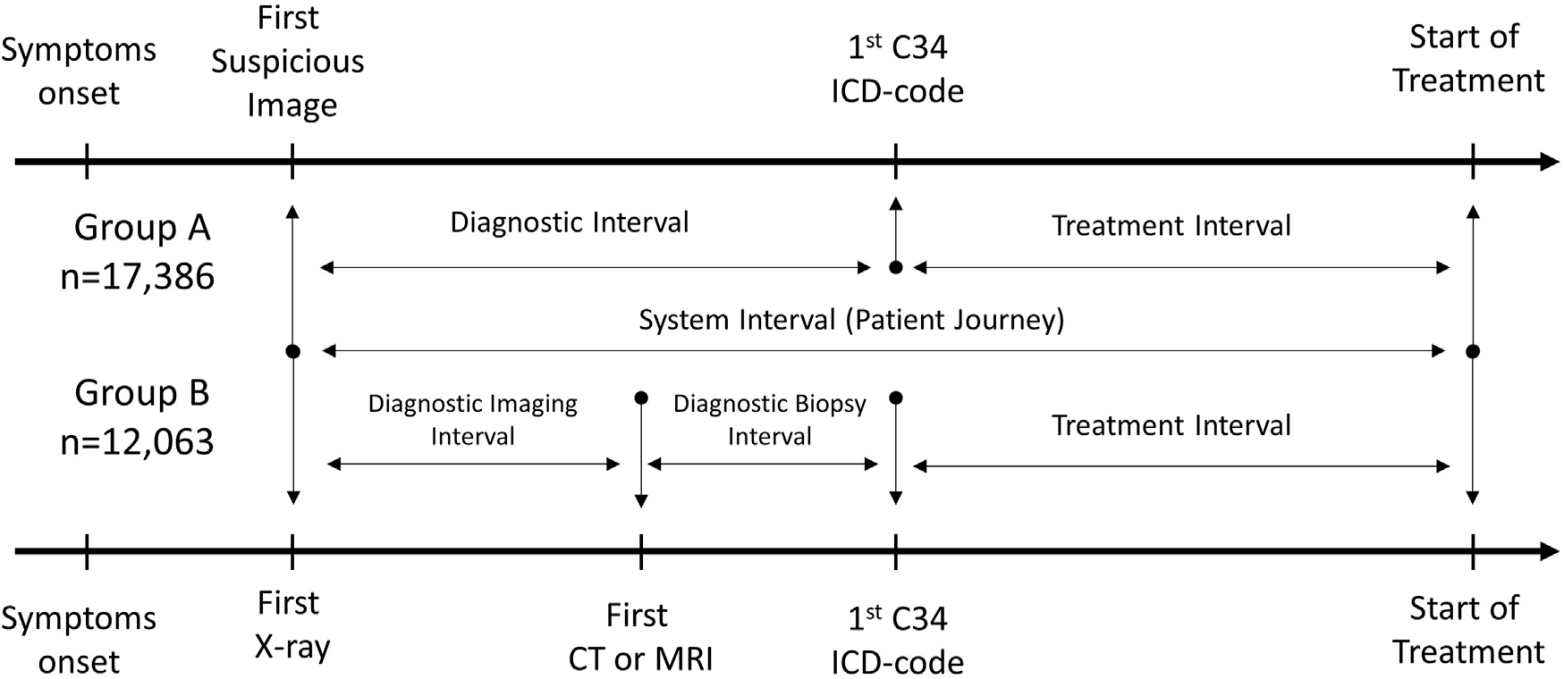
Milestones for defining diagnostic, treatment, and system intervals. The diagnostic interval is defined as the interval between the first suspicious image and the first record of C34; the treatment interval is defined as the period between the date of first C34 ICD code to the date of first-line therapy initiation; and the system interval is the sum of the diagnostic and treatment intervals. The diagnostic imaging interval is the period between the date of the first X-ray and the date of the first CT/MRI; the diagnostic biopsy interval is the period between the date of the first CT/MRI and the date of the first record of C34.

Figure 1 shows the milestones of the patient pathway analysed in our study. The *diagnostic interval* was defined as the interval between the first suspicious image and the first record of C34. To be more precise, the diagnostic interval was measured from the date of the first X-ray and CT/MRI (whichever occurred earlier within 120 days prior to the first C34 ICD code) until the date of the first C34 ICD code. The *treatment interval* was defined as the period between the date of the first C34 code record and the date of first-line therapy only if it was initiated within 90 days after the first C34 code. These periods were determined for group A (X-ray or CT/MRI plus first-line therapy codes) and group B (X-ray and CT/MRI plus first-line therapy codes). The *system interval* was the sum of the diagnostic and treatment intervals, i.e., the period from the date of the first suspicious image (first X-ray or CT/MRI, whichever occurred earlier) to the initiation of lung cancer therapy (only if it occurred within 90 days after the first C34 code), representing the whole patient pathway (Figure 1)**.** In Group B, where both X-ray and CT/MRI images were available along with records of first-line treatment, the diagnostic interval was divided into two further intervals: 1) *diagnostic imaging interval*: the period between the date of the first X-ray and the date of the first CT/MRI (within 120 days prior to the first C34 ICD code); 2) *diagnostic biopsy interval*: the period between the date of the first CT/MRI and the date of the first record of C34. The patient pathway was considered to start at the first outpatient specialist visit, therefore, previous symptoms and delays due to GP-related factors were out of scope of this analysis.

The length of intervals and the full length of the patient pathway were calculated in days. Group A served as the basis for overall system interval analysis. For the detailed analysis of the patient pathway, we calculated mean values of the diagnostic biopsy, diagnostic imaging, and treatment intervals in group B to examine their individual contribution to the whole patient pathway and potential changes over time. The mean, median and interquartiIe range (IQR) values of the various intervals were determined according to age, sex, study year, histology (where available), treatment type (chemotherapy, surgery, radiotherapy, and their combinations), and main Hungarian geographical regions. Median system intervals were compared using Wilcoxon’s rank-sum test in group A of lung cancer patients. The mean length of different intervals was compared using ANOVA (Analysis of Variance) in group B of lung cancer patients. The significance level was 5%. All calculations were performed with R version 3.5.2 (December 20, 2018) with package boot version 1.3-20.

## Results

Between January 1, 2011, and December 31, 2016, a total of 22,097 patients were registered in the NHIF database with a new lung cancer diagnosis and received any type of cancer therapy. X-ray or CT/MRI images were available for 17,386 of these patients prior to diagnosis (group A). 12,063 patients had records of both types of diagnostic imaging (X-ray and CT/MRI) before the record of the first C34 ICD-10 code (group B).

**FIGURE 2:**
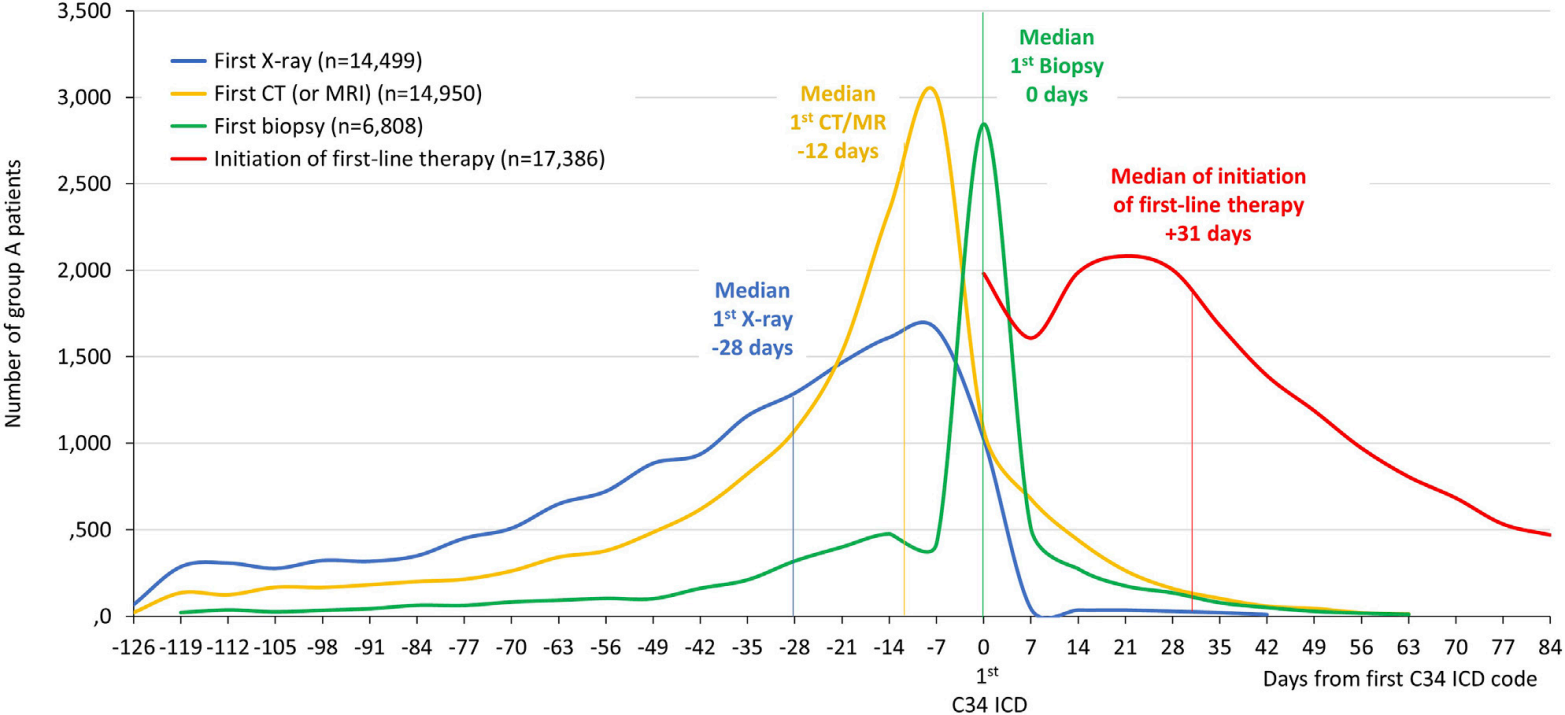
The distribution of the timing of X-ray, CT (or MRI), biopsy and initiation of first-line therapy in relation to the date of the first C34-ICD 10 code in group A patients.

The distribution of lung cancer patients as a function of the date of the first C34-ICD 10 code is shown in Figure 2 in group A. The median time between the first X-ray and first C34 ICD code was 28 days, 12 days between the first CT/MRI and C34, while the median of time difference between biopsy and C34 was zero. Therefore, we considered the date of the first C34 record as the date of the first biopsy for the rest of our analysis, even though we could not record the dates of biopsy in most cases as this intervention mostly happened in in-patient settings without separate records. The median of time difference between the first C34 ICD code and the date of initiation of first-line lung cancer treatment was 31 days.

### Analysis of System Interval in Group a of Lung Cancer Patients

**FIGURE 3:**
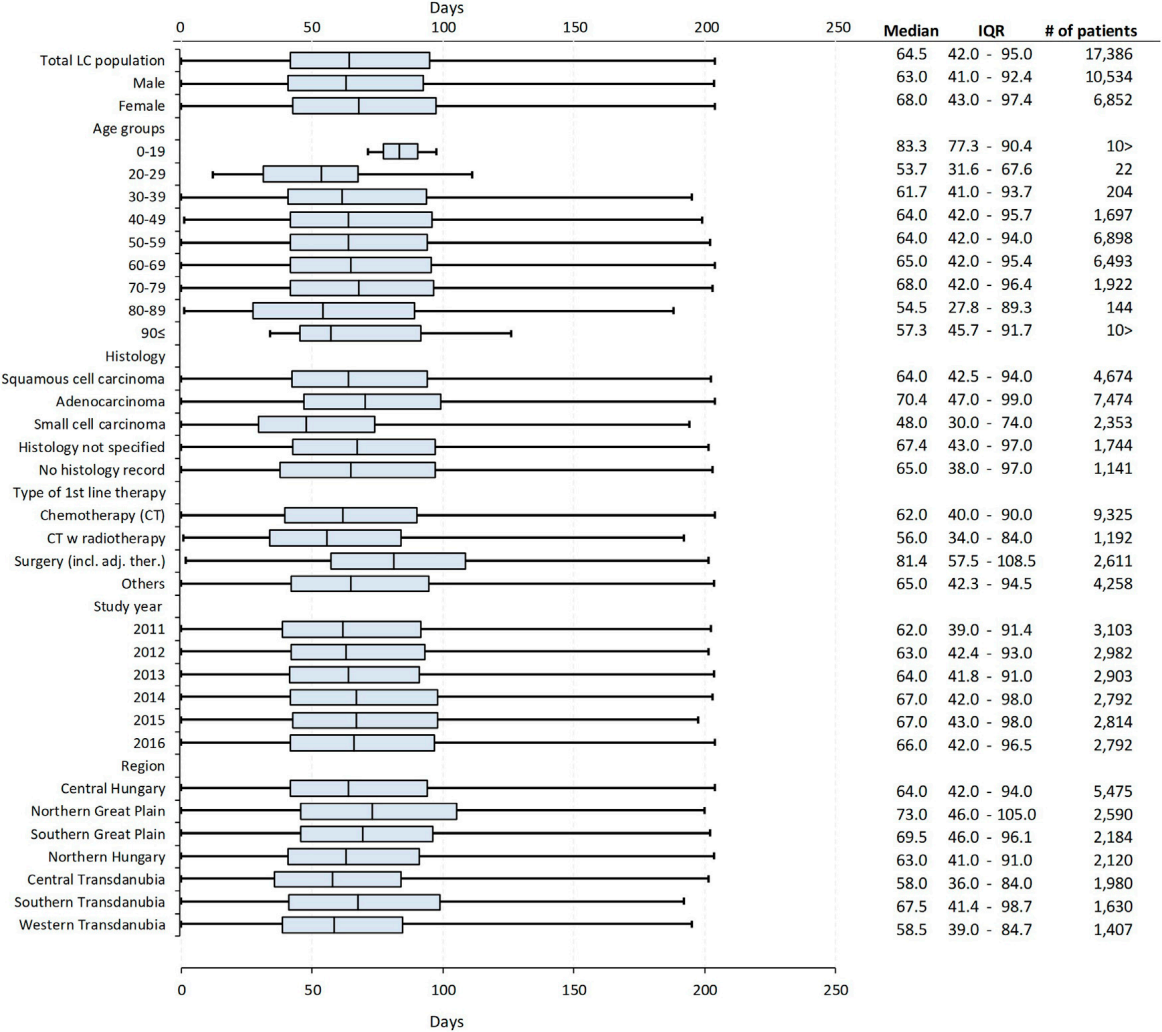
Median system intervals (the interval between the date of the first suspicious image and the date of the initiation of first-line therapy) in group A lung cancer patients by sex, age, histology of lung cancer, type of first-line therapy, study year, and main Hungarian regions, detailing the median, IQRs, number of patients with mean age (with SD) of the analysed subgroups.

Within group A of lung cancer patients, 60.59% of patients were male, the mean age at diagnosis was 62.39 years (SD ± 8.50) for all patients, 62.71 years (SD ± 8.28) for males and 61.89 years (SD ± 8.80) for females (Figure 3). The mean system interval from the first image to the start of treatment was 70.65 days (95% CI: 70.09–71.22) and the median was 64.5 days (IQR: 42–95) as shown in Supplementary Figure S1 and Figure 3. The median system interval was 5 days longer for females than for males (68.0 days; IQR: 43–97.4 vs. 63.0 days; IQR: 41–92.4; *p* < 0.001). No significant differences were found in the median system interval according to age groups.

The median system interval was significantly, 6.4 days longer in patients with adenocarcinoma (n = 7,474) compared to those with squamous cell carcinoma (n = 4,674) [70.4 days (IQR: 47–99) vs. 64.0 days (IQR: 42.5–94; *p* < 0.001), respectively]. As for histology, the shortest median system interval was recorded among patients diagnosed with small cell lung cancer [n = 2,353; median: 48.0 days (IQR: 30–74)], which was 22.4 days shorter than the period found in patients with adenocarcinoma (*p* < 0.001). A significantly longer system interval was detected among patients who had surgery as first-line treatment compared to those receiving chemotherapy [81.4 days (IQR: 57.5–108.5) vs. 62.0 days (IQR: 40–90) *p* < 0.001]. The median system interval increased from 62.0 days (IQR: 39–91.4) to 66.0 days (IQR: 42–96.5) during the 6-years study period, the difference was significant (*p* < 0.001). Furthermore, a significant difference was found between geographical regions: the shortest median system interval was found in Central Transdanubia and the longest in the Northern Great Plain region of Hungary [58.0 days (IQR: 36–84) vs. 73.0 days (IQR: 46–105); *p* < 0.001] (Figure 3).

### Detailed Patient Pathway Analysis in Group B of Lung Cancer Patients

**FIGURE 4:**
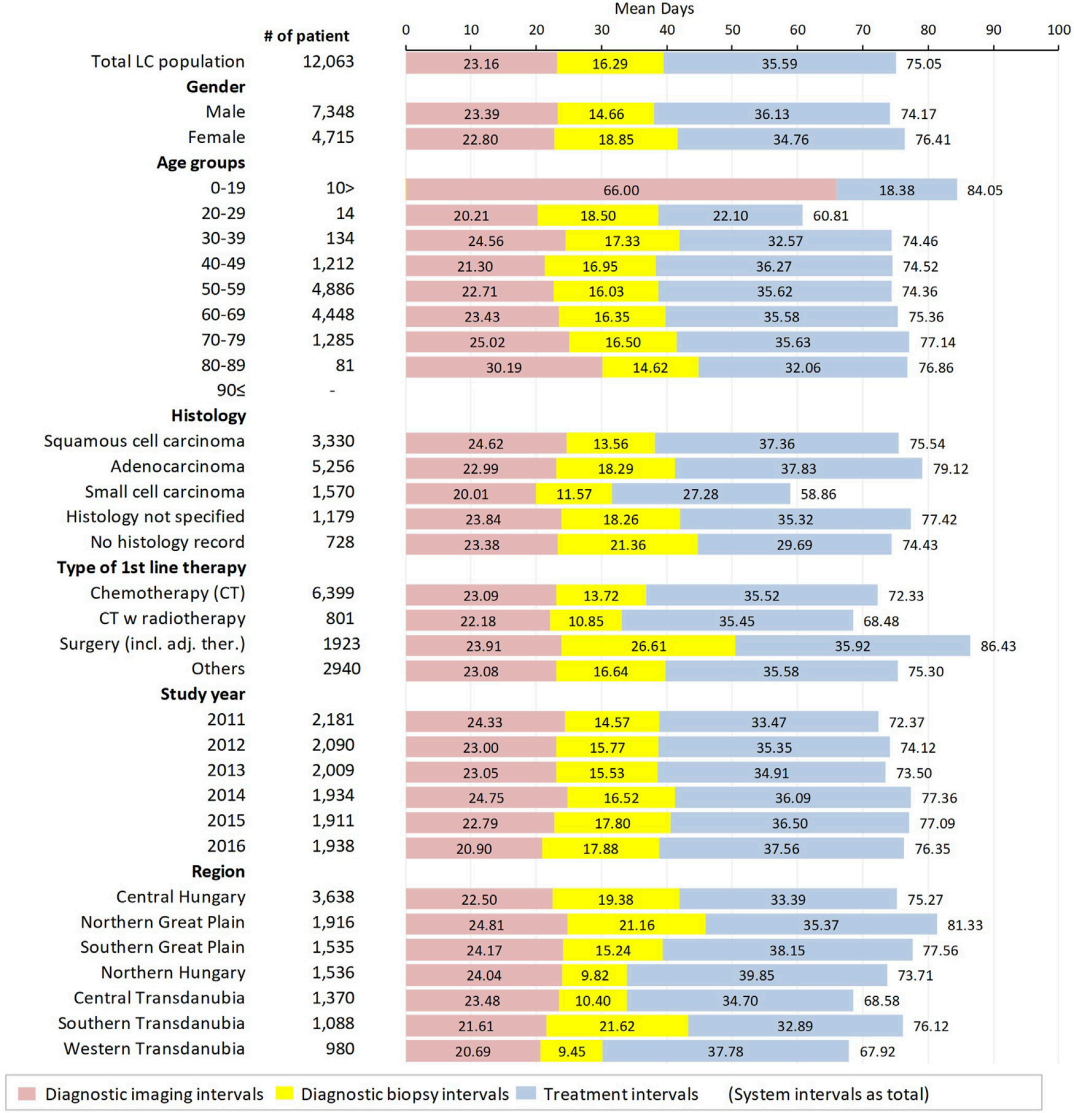
Mean diagnostic imaging intervals (period between the date of the first X-ray to the date of the first CT/MRI), diagnostic biopsy intervals (period between the date of the first CT/MRI to the date of the first record of biopsy), treatment intervals (period between the date of the first C34 ICD code to the date of first-line therapy initiation) and system intervals (sum of the diagnostic and treatment intervals–period between the first suspicious image and the first day of therapy) in group B lung cancer patients.

In group B lung cancer patients (n = 12,063), records of both X-ray and CT/MRI were available along with data regarding the initiation of first-line therapy. Therefore, in this group, we calculated *diagnostic imaging intervals* (from X-ray to CT/MRI) and *diagnostic biopsy intervals* (from CT/MRI to C34), as well as *treatment intervals* (from C34 to date of initiation of first-line therapy) and *system intervals* (from X-ray to the date of initiation of first-line therapy). Of note, the system intervals was not the mathematical sum of separate intervals but was calculated as the mean of the total length of the patient journey (from the first X-ray to the initiation of first-line therapy) (Figure 4).

Although the mean diagnostic imaging interval and the mean treatment interval were slightly, but not significantly shorter in females than males, the significantly longer mean system interval (+2.24 days in females; *p* < 0.001) was due to the significantly longer mean diagnostic biopsy interval (+4.19 days; *p* < 0.001). There was no significant difference in mean system intervals between different age cohorts, however, the mean diagnostic imaging interval showed a tendency to increase with age, from 20.21 to 30.19 days. Mean diagnostic imaging intervals were 1.63 days (*p* < 0.001) longer for squamous cell carcinoma and 2.99 days (*p* < 0.001) shorter for small cell carcinoma patients compared to patients with adenocarcinoma. Mean diagnostic biopsy intervals were 4.73 and 6.72 days shorter (*p* < 0.001 in both cases) for patients with squamous cell carcinoma and small cell carcinoma, respectively, compared to those with adenocarcinoma. The mean treatment interval was significantly shorter for patients with small cell carcinoma (27.28 days; 95% CI: 26.15–28.41) compared to patients with adenocarcinoma (37.83 days; 95% CI: 37.21–38.45), with a difference of −10.55 days (*p* < 0.001). On the other hand, there was no significant difference between the treatment intervals of patients with adenocarcinoma and squamous cell carcinoma (difference: −0.47 days, *p* = 0.350).

Patients undergoing surgery had significantly longer mean system intervals compared to those receiving chemotherapy as first-line treatment (86.43 days; 95% CI: 84.84–88.02 vs. 72.33 days; 95% CI: 71.46–73.20), with a difference of 14.10 days (*p* < 0.001) which was mostly due to significantly longer mean diagnostic biopsy intervals (12.89 days; *p* < 0.001). The significantly shorter mean system interval observed among patients receiving radiotherapy + chemotherapy compared to patients receiving only chemotherapy (−3.85 days; *p* < 0.001) was driven by longer mean diagnostic biopsy intervals in the latter patient group (difference: 2.87 days; *p* < 0.001).

The mean length of the whole patient pathway significantly increased by almost 4 days (3.98 days; *p* < 0.001) from 2011 to 2016, which was the result of two opposite tendencies. On one hand, the mean diagnostic imaging interval decreased by 3.43 days from 24.33 to 20.90 days (*p* < 0.001), on the other hand, the mean diagnostic biopsy interval increased by 3.31 days (*p* < 0.001), and the mean treatment interval also increased by 4.09 days (*p* < 0.001) during the 6-years study period.

There was a more than 13.41-days difference in the mean length of the whole lung cancer patient pathway between the main Hungarian regions. The longest mean diagnostic imaging interval was found in the Northern Great Plain, which was 2.31 days longer than in the Central Hungarian region (*p* < 0.001), while the longest mean diagnostic biopsy interval was detected in Southern Transdanubia (21.62 days; 95% CI: 23.11–20.13). However, the shortest mean treatment interval was recorded in Southern Transdanubia (32.89 days; 95% CI: 31.52–34.26). The longest mean treatment interval was in Northern Hungary (39.85 days; 95% CI: 38.70–41.01), which was 6.46 days longer than in the Central Hungarian region.

Median diagnostic imaging intervals, diagnostic biopsy intervals, treatment intervals and system intervals in group B lung cancer patients according to sex, age, histology of lung cancer, type of first-line therapy, study year, and main Hungarian regions are shown in Supplementary Table S2.

## Discussion

This study was the first to describe the characteristics of the lung cancer patient pathway in Hungary and to examine changes in the length of the whole patient pathway and its individual components over time. The main findings of our nationwide, retrospective, study involving more than 20,000 patients over 6 years can be summarized as follows:

1) The median system interval, i.e., the length of the whole lung cancer patient pathway was 64.5 days (group A). No significant differences were observed according to age; however, female sex, adenocarcinoma, and surgery as first-line treatment were associated with significantly longer median system intervals.
2) We found significant changes in the length of the system interval as well as its individual components during the 6-years study period (group A and group B). The median system interval increased by almost 4 days from 2011 to 2016 (group A), which was mainly driven by increases in the diagnostic biopsy interval and treatment interval. However, we found a more than 3-days decrease in the diagnostic imaging interval between 2011 and 2016.
3) Significant differences were found between the main Hungarian regions, suggesting that there are relevant differences in the access to medical care along the patient pathway between different regions (both in group A and group B).

### Results of System Interval Analysis in the Context of Previous Findings

Detecting lung cancer at an early stage is challenging, and the resulting delays in diagnosis may adversely affect survival. Therefore, optimizing the patient pathway is crucial for improving patient outcomes (12). Recognizing the importance of delays along the patient pathway, several consensus-based standards have been established during the past decades regarding maximum acceptable waiting times for the referral, diagnosis, and treatment of lung cancer. In 1998, the British Thoracic Society (BTS) guidelines recommended a maximum period of 70 days between radiography and thoracotomy (13). A few years later, the RAND Corporation recommended that treatment be initiated within 42 days of diagnosis and that the total time between the initial abnormal radiographic image and treatment initiation should not exceed 98 days (14). The Swedish Lung Cancer Study Group developed even stricter recommendations and suggested that treatment be initiated within 42 days of suspicious imaging (15). In 2011, the National Institute for Health and Care Excellence (NICE) published its revised guidelines on lung cancer care, diagnosis, and treatment, which included a recommendation for diagnostics stating that patients with suspected lung cancer should receive a specialist appointment within 14 days and that X-rays be performed within 14 days for patients meeting certain clinical criteria for lung cancer risk (16).

In our study, the median time from the first suspicious image to the initiation of lung cancer therapy, i.e., the system interval was 64.5 days, which is largely consistent with international observations. A Canadian study reported a slightly longer median system interval of 78 days in patients with non-small cell lung cancer, while a recent Finnish study recorded a shorter median system interval of 38 days, with a median of 23 days from referral by a primary care physician to pathologic diagnosis and a median time of 15 days from diagnosis to treatment initiation (17,18). A U.S. study examined NSCLC patient waiting times and found an 82-days median system interval from the detection of a pulmonary lesion to surgical intervention (19). Furthermore, a study by Yorio et al. found a median overall image-treatment interval of 59 days, which comprised the evaluations and planning required for the imaging and diagnosis-treatment intervals. In this report, the 25–75% IQR was 34–93 as opposed to 42–95 in our analysis, suggesting that LC patients may receive treatment sooner after the first suspicious image in the U.S., than in Hungary (20). In 2017, Jacobsen et al. reviewed 65 studies from 21 different countries to examine the timeliness of lung cancer care and found significant variations in the reporting of intervals related to lung cancer diagnosis and treatment. However, the authors were able to identify the time between the initial suspicious image of lung cancer and the first day of treatment (i.e., the system interval) in most studies, with reported medians ranging from 71 to 117 days (21). The authors also highlighted the difficulties to compare parts of system intervals across different reports, hence the importance of establishing standardized wait-interval metrics to evaluate and improve the timeliness of lung cancer diagnosis and treatment.

Although our results regarding the length of the system interval are in line with previous investigations, we found a significantly longer system interval among females than in males, with no significant differences according to age. In contrast, in the study by Yorio et al., the median system interval was associated with race, income, and age, and although it was longer in women than in men, the difference was not significant (20). However, this study was conducted in a much smaller population of LC patients, and different age cohorts were used. A larger population of LC patients by Kim et al. found that the risk of delayed diagnosis consistently increased with age, with patients over 80 having the highest risk of delayed diagnostic intervals. However, gender, comorbidities and socioeconomic status were not associated with delayed system intervals (17). Further studies are needed to explore potential reasons for these differences, and to identify patient populations at a higher risk of delays along the patient pathway as well as factors responsible for these delays.

Apart from age and sex, a number of other patient characteristics have been associated with longer waiting times. Several studies have demonstrated a positive association between a lower level of education and longer delays along the patient pathway (22,23,24), while others found no association between education and delays in diagnosis and treatment (20). Furthermore, a number of studies suggest that there may be geographic differences in LC patient pathway waiting times within the same country, with the majority of studies reporting longer delays among patients living in rural areas than in urban patients (25,26,27)*.* In line with these observations, we found significant differences in median system intervals between the main regions of Hungary: the shortest median system interval was found in Central Transdanubia, and the longest in the Northern Great Plain. Since our analysis did not account for the time before the first presentation to the GP, the potential reasons for geographic inequalities in the LC patient pathway warrant further investigation. Nevertheless, the results call the attention of the healthcare government to better resource allocation within the country.

The histology of lung cancer and the type of first-line therapy have also been shown to influence the length of the patient pathway (17). In our study, patients with adenocarcinoma had a longer median system interval (70.4 days), than those with squamous cell carcinoma (64 days) or small cell lung cancer (48 days). These results are in line with findings by Kim et al., where patients with adenocarcinoma had a longer waiting time to treatment than patients with squamous cell carcinoma (17)*.* The comparatively shorter median system interval found in patients with small cell lung cancer may be explained by the rapid progression of this type of LC and the resulting greater clinical urgency of treatment initiation which may motivate quicker decision-making regarding therapy. In this aspect, the increasing incidence of adenocarcinoma and the increasing contribution of women to LC incidence reported in our previous publication on LC survival within the same period (28) may also contribute to increases in the length of the patient pathway. In addition, the increasing incidence of adenocarcinoma may also increase the length of the diagnostic interval, as the peripheral location of this tumor type may lead to more complicated biopsy procedures.

In a recent study by Kim et al., patients scheduled for surgical treatment had to wait 44% longer from diagnosis to treatment than those who received radiotherapy or chemotherapy as first-line treatment. Furthermore, surgical patients had the highest risk for delayed treatment, and the longest total diagnostic (diagnostic imaging and diagnostic biopsy) interval, treatment interval, and system interval (17)*.* In line with these findings, we found a significantly longer median system interval among patients having surgery as first-line treatment compared to those receiving chemotherapy, with a difference of almost 20 days. Previous reports exploring the potential reasons for this phenomenon generally concluded that the need for comprehensive preparations such as cardiopulmonary assessments prior to surgery compared with other forms of first-line therapy may be responsible for longer waiting times among patients undergoing surgery (17,29,30)*.* Therefore, straightforward recommendations and well-coordinated patient pathways are vital for the optimization of pre-surgery intervals within the patient pathway (29,30)*.*

### Details of the Patient Pathway

To identify the contribution of specific intervals to the whole patient pathway and their relations to each other, we examined the individual components of the system interval among patients who had available data regarding X-ray, CT/MRI, and the initiation of first-line therapy. Although these parts of patient pathways are usually presented in medians, we expressed both means and medians in Figure 4 and Supplementary Table S1 to allow for comparisons with international findings as well as across different subgroups. We found that the longest part of patient pathway was the treatment interval (median: 32.54 days and mean: 35.59 days) followed by the diagnostic imaging interval (median: 16.0 days and mean: 23.16 days) and the shortest period was the diagnostic imaging interval (median: 11.0 days and mean: 16.29 days).

A study by Kim et al. conducted in Canada between 2004 and 2011 found a median diagnostic imaging interval of 10 days, a median diagnostic biopsy interval of 19 days, and a median total diagnostic interval of 38 days (17). Schultz et al. reported a 33-days median for the total diagnostic interval of NSCLC patients in the U.S. between 2002 and 2005 (31), while Faris et al. reported 28 days for the 2009–2013 period (32). Verma et al. reported a mean total diagnostic interval of 27 days for NSCLC patients between 2013 and 2014 (33). Overall, lung cancer was diagnosed within 28–38 days from the first suspicious image. In Hungary, we found a median total diagnostic interval of 31.0 days (IQR: 16–50) between 2011 and 2016, which is comparable with previous results.

Based on a 2015 decree of the Hungarian Ministry of Human Resources (1/Jun/2015) (34), the time from suspicious X-ray to CT or MRI imaging should not exceed 14 days, meaning that patients for whom X-ray findings raise the suspicion of LC should have access to CT (or MRI) within 14 days. Between 2011 and 2016, 50% of patients had access to CT imaging within a median of 16 days (IQR 25%: 8.00; IQR 75%: 23.16). This period significantly decreased during the study period and reached 13.0 days by 2016 (IQR: 7.0–27.0), meeting the target established by the Ministry of Human Resources (EMMI) on a national level.

On the other hand, the diagnostic biopsy and treatment intervals increased between 2011 and 2016. The median diagnostic biopsy interval was 10.0 days in 2011 (mean: 14.57 days) and increased by 2 days (*p* < 0.001) by 2016 (mean change: 3.31 days). This increase was also observed in the treatment interval, where the median changed from 30.0 to 35 days (*p* < 0.001) (mean from 33.47 to 37.56 days) during the study period. Kim reported a 14% increase in the delay of system intervals between 2004 and 2011, although this delay was not significant most probably due to the small size of the examined lung cancer population. To our knowledge, our study was the first to show increases in LC patient pathway intervals over time. The large size of our study population provided good basis for the detailed investigation of this change and revealed significant differences between study years. We assume that increases in diagnostic (biopsy) and treatment intervals during the 6-years study period may be due to the increasing number and more specific diagnostic steps, such as molecular pathology, which requires more time, but may be correlated with better outcomes. This assumption is supported by a recent publication which showed a 9% increase in the 5-years survival rate of Hungarian LC patients during the same, 2011–2016 study period, demonstrating that more specific diagnostic procedures take more time but result in better survival.

Our analysis showed that the significantly longer system intervals observed in women vs. men (median: 2.5 days and mean: 2.24 days; both *p* < 0.001) mostly originated from the increase in diagnostic biopsy intervals (median: 3.0 days and mean: 4.19 days; both *p* < 0.001) which exceeded the slight decrease in treatment intervals. Kim et al. did not find any significant difference in these periods between females and males (17), and Vinod could not provide evidence for the impact of gender on treatment delay (7). We assume that the longer system interval of female patients may be attributed to the higher proportion of adenocarcinoma in women which is associated with a longer diagnostic and consequently a longer system interval.

We found a slight increase in the system interval with age, which mostly originated from the increase in the diagnostic imaging interval, suggesting that elderly lung cancer patients have slower access to CT (or MRI) imaging after a suspicious X-ray image. On the other hand, we found an age-related decrease in the treatment interval. Kim et al found 44–73% more delays in the system interval in the age group of ≥60 vs. <60 years, with similar delays in the diagnostic and treatment intervals (17). On the other hand, Yorio et al. found no significant differences in image–diagnosis intervals and diagnosis–treatment intervals, neither in image–treatment intervals in patients aged ≥65 vs. <65 years (20). We assume that younger age at the time of suspicious X-ray findings may urge healthcare providers to confirm the findings with more specific CT imaging, hence leading to shorter diagnostic imaging intervals.

There was a 5.37-day difference in the length of the total patient pathway between patients with adenocarcinoma and squamous cell carcinoma, although differences in individual parts of the patient pathway were not relevant. On the other hand, the significantly shorter system interval in patients with small cell carcinoma (−24.0 days median compared to squamous cell carcinoma) mostly originated from significantly shorter diagnostic imaging (−5 days) and treatment (−12.42 days) intervals as small cell carcinoma requires a less detailed diagnostic workup but urges faster therapy initiation (33).

The diagnostic imaging interval showed significant variations across main Hungarian regions, with a 5-days difference in median between the Southern Great Plain [median: 19.0 (IQR: 8.0–35.0)] and Western Transdanubia [median: 14.0; (IQR: 7.0–26.0)]. In addition, the imaging biopsy interval was the highest in the Northern Great Plain and in Southern Transdanubia (medians 2.0–4.0 days longer vs. Central Hungary). The treatment interval was the shortest in Central Hungary (median 8 days shorter vs. Northern Hungary). These results highlight the importance of the regular evaluation of disparities in cancer management as well as the planning of better resource allocation for improving the outcomes of lung cancer strategy. The regional differences in mean treatment intervals could be partially explained by the fact that in Hungary, lung cancer diagnosis and treatment are often still carried out in remote, isolated sanatoriums once built for the chronic care of tuberculosis patients, instead of multidisciplinary medical centers. Further studies are needed to explore the potential reasons behind significant differences in different intervals between the main Hungarian regions to help optimize the timing of treatment initiation for the whole Hungarian LC patient population. In addition, further studies may reveal potential associations between the length of patient pathway components and lung cancer survival.

Our study has certain strength and limitations. The robust number of investigated lung cancer patients and the length of the study period provided a strong basis for the evaluation of individual parts of the LC patient pathway, as well as for subgroup analyses. In this aspect, our study is one of the largest in the past decade examining the LC patient pathway. On the other hand, inpatient X-ray or CT/MRI examinations could not be identified in the NHIF database, which limited the detailed investigation of the patient pathway in a population of 4,711 patients who received first-line LC treatment without NHIF records on diagnostic imaging. The same applied to the date of biopsy, for which 6,808 patients with X-ray and/or CT/MRI records did not have any data recorded. Nevertheless, we solved this problem by dividing the study population into three subgroups based on the availability of information. Moreover, the NHIF database does not contain any data on the staging or ECOG status of patients, and no laboratory test results were available. Consequently, we were not able to provide specific evaluation in this aspect in the lung cancer population. In addition, the investigated 6-years study period ended 5 years ago, however, this is the first detailed patient pathway analysis of a nationwide lung cancer population in Hungary, and the applied methodology could serve as a basis for future patient pathway analyses.

## Conclusion

This nationwide retrospective study provides valuable insights into the characteristics of the lung cancer patient pathway in Hungary and its changes over a 6-years period. The length of the whole patient pathway was comparable to reports from other countries and showed a 4-days increase during the study period, mainly due to longer treatment intervals associated with more specific examinations. These examinations eventually contributed to the 9% improvement in 5-years LC survival recently published for the same lung cancer population.

The length of the whole patient pathway was significantly longer among female vs. male patients. In line with previous observations, surgery as first-line treatment was associated with a longer time-to-treatment interval compared to other treatment modalities, however, the length of the patient pathway was not significantly influenced by age. The significant regional differences observed within Hungary indicate the need for allocating resources to areas with longer waiting times or worse access to medical care. The detailed analysis of the patient pathway allowed for the detection of temporal trends as well as disparities among patient subgroups, highlighting that there is still room for improvement to optimize the timeliness of lung cancer care. Although the association between the total length of the LC patient pathway and LC survival is an area of controversy (35) resulting in a “waiting times paradox” (17), delays in diagnostic phases may lead to survival loss, therefore, LC patients could benefit from improvements in the first parts of the patient pathway. This is particularly important in 2020 and 2021 in view of the Covid-19 pandemic. A new local study investigating the impact of the pandemic on the LC patient pathway is currently underway.

## Data Availability

The raw data supporting the conclusions of this article will be made available by the authors, without undue reservation.

## Supplementary Material

Supplementary Figure S1 Mean values of system intervals in group A lung cancer patients according to sex, age, histology type, treatment type, study years and the main Hungarian regions.
