# Supplementary material for: Increase in the Length of Lung Cancer Patient Pathway Before First-Line Therapy: A 6-Year Nationwide Analysis From Hungary
Source: Pathol Oncol Res. 2021 Dec 23;27:1610041. doi: 10.3389/pore.2021.1610041 (PMC8734146; doi:10.3389/pore.2021.1610041)
Supplement: Supplementary file 1 [file Table1.docx]

**Supplementary table 1**

**12.1 Definition of cancer treatment with ICPM codes (based on Hungarian OENO definition):**

7099 Chemotherapy according to TAX + CBP protocol (carboplatin paclitaxel)

7052 Chemotherapy, GEM + CDDP II. protocol (cisplatin gemcitabine)

7092 Chemotherapy according to CBP + VP protocol (carboplatin etoposide)

7180 Chemotherapy according to GEM + CBP / B protocol (carboplatin gemcitabine)

7184 Chemotherapy, TXT / B monotherapy protocol (docetaxel)

7097 Chemotherapy according to PE protocol (cisplatin etoposide)

7185 Chemotherapy, PEM monotherapy protocol (pemetrexed)

7191 Chemotherapy according to PEM-CDDP protocol (cisplatin pemetrexed)

7474 Chemotherapy according to TAX + CBP + BEV protocol (bevacizumab carboplatin paclitaxel)

7186 Chemotherapy according to CDDP + VNB / B protocol (cisplatin vinorelbine)

7059 Chemotherapy according to TXT + CDDP protocol (cisplatin docetaxel)

7077 Chemotherapy according to ECO protocol (cyclophosphamide epirubicin vincristine)

7362 Chemotherapy, BEV lung monotherapy protocol (bevacizumab)

7187 Chemotherapy according to VNB protocol (vinorelbine)

7053 Chemotherapy according to GEM / B protocol (gemcitabine)

7179 Chemotherapy according to GEM / C protocol (gemcitabine)

7188 Chemotherapy according to PE / B protocol (cisplatin etoposide)

7367 Chemotherapy according to TOPO / B protocol (topotecan)

7183 Chemotherapy according to CBP + TXT / C protocol (carboplatin docetaxel)

7369 Chemotherapy according to CDDP + TAX / B protocol (cisplatin paclitaxel)

7921 Chemotherapy, TXT monotherapy protocol (docetaxel)

7458 Chemotherapy according to VNB + CBP protocol (carboplatin vinorelbine)

7181 Chemotherapy according to TAX + CBP / D protocol (carboplatin paclitaxel)

7423 Radiochemotherapy for non-small cell lung cancer according to TXT-CDDP protocol (cisplatin docetaxel radiotherapy)

7182 Chemotherapy according to CDDP + TAX / C protocol (cisplatin paclitaxel)

7193 Chemotherapy according to ECO / B protocol (cyclophosphamide epirubicin vincristine)

7920 Chemotherapy, TAX monotherapy protocol (paclitaxel)

7076 Chemotherapy according to CDDP + VNB protocol (cisplatin vinorelbine)

7320 Chemotherapy according to GEM + CBP / C protocol (carboplatin gemcitabine)

7420 Radiochemotherapy for non-small cell lung cancer according to TAX protocol (paclitaxel radiotherapy)

7436 Chemotherapy according to GEM + CDDP II + BEV protocol (bevacizumab cisplatin gemcitabine)

7189 Chemotherapy according to PE / C protocol (cisplatin etoposide)

7424 Radiochemotherapy for non-small cell lung cancer according to TAX-CBP protocol (carboplatin paclitaxel radiotherapy)

7051 Chemotherapy according to GEM + CDDP Protocol I (cisplatin gemcitabine)

7477 Chemotherapy according to CDDP + TAX / C + BEV protocol (bevacizumab cisplatin paclitaxel)

7419 Radiochemotherapy for CDDP according to CDDP for lung cancer (cisplatin radiotherapy)

7422 Radiochemotherapy for CDDP-VP / B in lung cancer (cisplatin etoposide radiotherapy)

7456 Chemotherapy, IRE monotherapy according to protocol (gefitinib)

7437 Chemotherapy according to TXT + CDDP + BEV protocol (bevacizumab cisplatin docetaxel)

7475 Chemotherapy according to GEM + CBP / B + BEV protocol (bevacizumab carboplatin gemcitabine)

7478 Chemotherapy according to CBP + TXT / C + BEV protocol (bevacizumab carboplatin docetaxel)

7094 Chemotherapy according to CEV (small cell lung cancer) protocol (cyclophosphamide epirubicin etoposide)

7050 Chemotherapy according to GEM / A protocol (gemcitabine)

7091 Chemotherapy according to CAV protocol (cyclophosphamide doxorubicin vincristine)

7054 Chemotherapy, GEM + CDDP III. protocol (cisplatin gemcitabine)

7421 Radiochemotherapy for lung cancer according to CDDP-VP / A protocol (cisplatin etoposide radiotherapy)

7438 Chemotherapy according to CDDP + VNB / A protocol (cisplatin vinorelbine)

7342 Chemotherapy according to TOPO / A protocol (topotecan)

7157 Chemotherapy according to GEM + CBP protocol (carboplatin gemcitabine)

7167 Chemotherapy according to TAX + CBP / B protocol (carboplatin paclitaxel)

7162 Chemotherapy according to TOPO / C protocol (topotecan)

7468 Radiochemotherapy for CBP according to CBP protocol (carboplatin radiotherapy)

7190 Chemotherapy according to CBP + VP / B protocol (carboplatin etoposide)

7196 Chemotherapy according to VNB / A protocol (vinorelbine)

**12.2 Definition of LC surgery with ICPM codes:**

53240    Lobectomia   superior

53242    Lobectomia   inferior

54020    Lymphadenectomia regionalis

53230    Resectio atypica laesionis pulmonis

53251    Pneumonectomia radicalis

53253 Pneumonectomy (extended in 1 direction)

53256 Pneumonectomy (multidirectional extension)

53241    Lobectomia   media

53244    Bilobectomia inferior

53237    Segmentectomia pulmonis

53243    Bilobectomia superior

53250    Pneumonectomia sec. Allison

53245    Lobectomia   superior et sleeve resectio

53233    Csonkalebeny resectio (lung)

**12.3 Definition of radio-therapy with ICPM codes:**

38621 HDR AL treatment, bronchus (one way)

38622 HDR AL treatment, bronchus (multipath)

**12.4 Definition of palliation radio-therapy with ICPM codes:**

37283 Stereotaxic cerebral irradiation per focal (photon)

37284 Stereotaxic cerebral radiosurgery, first target

37285 Stereotaxic cerebral radiosurgery, additional target

37288 Brain gamma surgery

37520 Cobalt-60 telotherapy, one field
