# Supplementary figures and images for: Increase in the Length of Lung Cancer Patient Pathway Before First-Line Therapy: A 6-Year Nationwide Analysis From Hungary
Source: Pathol Oncol Res. 2021 Dec 23;27:1610041. doi: 10.3389/pore.2021.1610041 (PMC8734146; doi:10.3389/pore.2021.1610041)

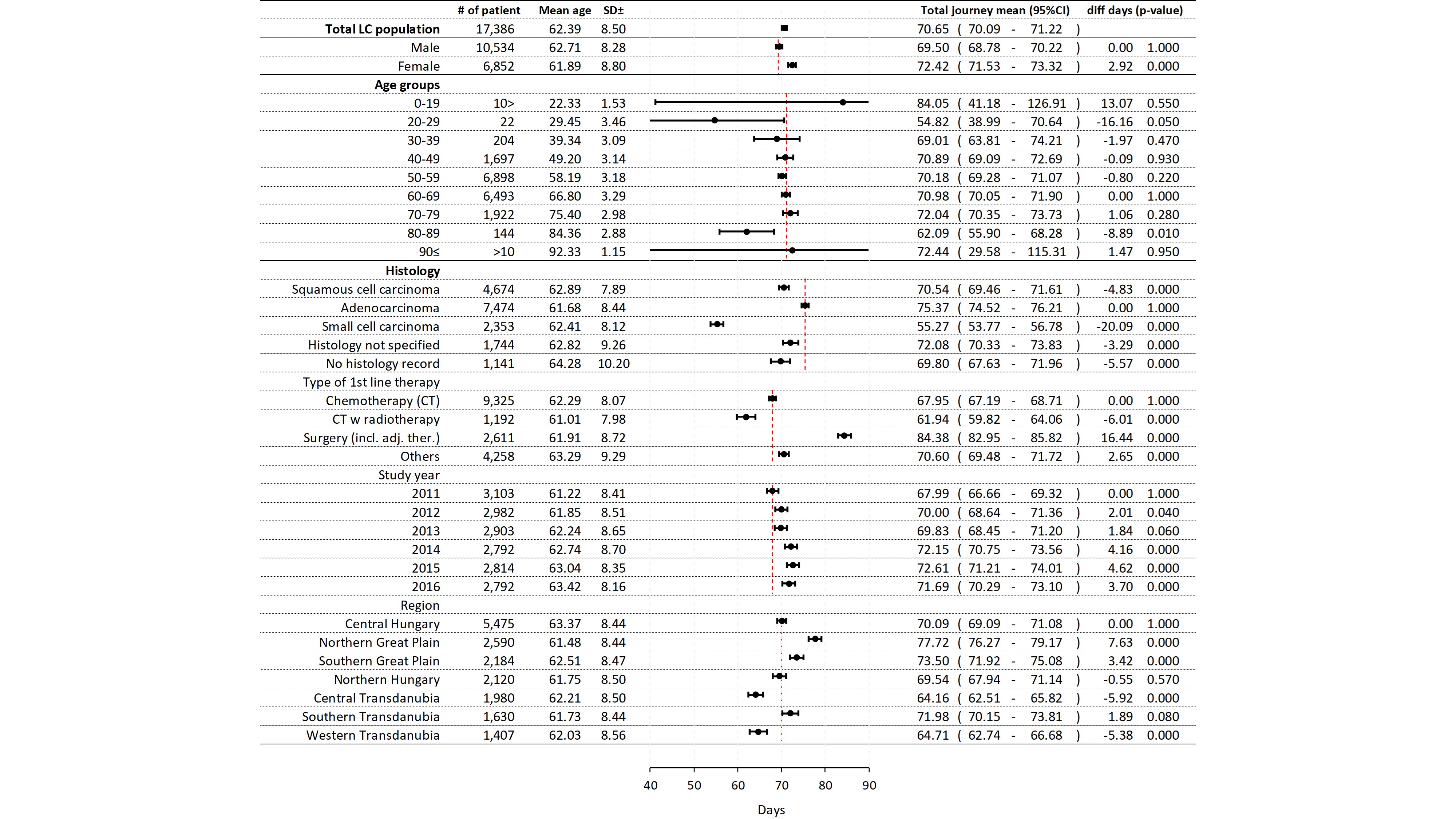

Supplement: Supplementary file 2 [file Image1.JPEG]
