# Supplementary material for: Increase in the Length of Lung Cancer Patient Pathway Before First-Line Therapy: A 6-Year Nationwide Analysis From Hungary
Source: Pathol Oncol Res. 2021 Dec 23;27:1610041. doi: 10.3389/pore.2021.1610041 (PMC8734146; doi:10.3389/pore.2021.1610041)
Supplement: Supplementary file 3 [file Table2.doc]

**Supplementary Table 2** Median diagnostic imaging intervals, diagnostic biopsy intervals, treatment intervals and system intervals in group B lung cancer patients according sex, age, histology of lung cancer, type of first-line therapy, study year, and main Hungarian regions
